# ESWT attenuates compressive stress-induced cartilaginous endplate degeneration via modulation of the *SOST/Wnt/β-catenin* signaling pathway

**DOI:** 10.3389/fbioe.2026.1769920

**Published:** 2026-06-24

**Authors:** Pan Xiang, Junjun Hou, Chao Yang, Ting Liang, Wenbin Cai, Fanlei Yang, Jing Yang, Hao Xu, Zongping Luo, Yanjun Che

**Affiliations:** 1 Department of Orthopaedics, The First Affiliated Hospital of Soochow University, Suzhou, Jiangsu, China; 2 Institute of Orthopedics, Soochow University, Suzhou, Jiangsu, China; 3 Department of Endocrinology, The First Affiliated Hospital of Soochow University, Suzhou, Jiangsu, China; 4 Department of Geriatrics, Xinghu Hospital, Suzhou, Jiangsu, China; 5 Department of Clinical Nutrition, The First Affiliated Hospital of Soochow University, Suzhou, China; 6 Orthopedics and Sports Medicine Center, The Affiliated Suzhou Hospital of Nanjing Medical University, Suzhou, Jiangsu, China; 7 Gusu School, Nanjing Medical University, Suzhou, Jiangsu, China; 8 Suzhou Municipal Hospital, Suzhou, Jiangsu, China

**Keywords:** cartilaginous endplate, extracorporeal shockwave therapy, intervertebral disc degeneration, mechanical stimulation, SOST, Wnt/β-catenin

## Abstract

**Introduction:**

Cartilaginous endplate (CEP) degeneration is a critical contributor to intervertebral disc degeneration (IVDD) and is frequently induced by sustained compressive stress. Extracorporeal Shockwave Therapy (ESWT) has shown therapeutic potential in promoting disc repair; however, the underlying molecular mechanisms remain unclear. This study aimed to investigate the role of sclerostin (SOST) and Wnt/β-catenin signaling pathway in compression-induced CEP degeneration and to explore the protective effects of ESWT.

**Methods:**

*In vivo*, a custom-designed compression device was applied to rat tails to establish a disc degeneration model. Histological staining, scanning electron microscopy, and radiological imaging were employed to evaluate disc morphology, endplate integrity, and degenerative changes. *In vitro*, CEP cells were subjected to mechanical compression followed by ESWT treatment. The expression levels of SOST, key components of the Wnt/β-catenin pathway, and extracellular matrix metabolism-related markers were assessed using qRT-PCR and Western blotting.

**Results:**

*In vivo*, ESWT improved disc height and MRI signal intensity, restored endplate structural integrity, upregulated SOST expression, and suppressed activation of the Wnt/β-catenin pathway compared with spontaneous recovery. *In vitro*, compressive stress markedly downregulated SOST expression in CEP cells, activated Wnt/β-catenin signaling, decreased chondrogenic marker expression, and increased the expression of catabolic and calcification-related genes. SOST overexpression alleviated these degenerative alterations.

**Conclusion:**

ESWT attenuates mechanical loading-induced CEP degeneration by upregulating SOST expression, which *in vitro* evidence suggests inhibits aberrant activation of the Wnt/β-catenin pathway and suppresses catabolic and calcification processes. These findings suggest that ESWT represents promising therapeutic strategy for CEP degeneration.

## Introduction

Cartilaginous endplate (CEP) degeneration is increasingly recognized as a pivotal initiating event in intervertebral disc degeneration (IVDD), a major pathological basis of chronic low back pain worldwide (Diseases and Injuries, 2020; Cieza et al., 2021). The vertebral endplate, encompassing both cartilaginous and osseous components, serves not only as the principal conduit for nutrient diffusion between the vertebral body and the intervertebral discs but also as a critical biomechanical interface. It withstands tensile and shear stresses generated by intradiscal pressure and the annulus fibrous, thereby ensuring uniform transmission and distribution of compressive loads to the adjacent vertebral bodies (Baranto et al., 2005). The coordinated function of the CEP and the bony endplate (BEP) is essential for maintaining disc homeostasis through the regulation of nutrient transport and mechanical load distribution. Abnormal mechanical loading can disrupt this delicate balance, triggering pathological changes such as CEP degeneration, BEP calcification, and altered annulus fibrosus (AF) tension, all of which accelerate IVDD progression. Our preliminary research has affirmed that the remodeling of the endplate’s mechanical microenvironment can restore degenerated intervertebral discs to a certain extent (Che et al., 2021; Che et al., 2022). Despite growing evidence linking aberrant mechanical stress to CEP degeneration (Zehra et al., 2015; Tomaszewski et al., 2015; Xia et al., 2015), the molecular mechanisms through which biomechanical cues are transduced into degenerative cellular responses remain poorly understood. Elucidating these mechanotransduction pathways is therefore essential for identifying effective therapeutic strategies targeting CEP degeneration and preventing IVDD progression.

Accumulating evidence indicates that aberrant activation of the Wnt/β-catenin signaling pathway plays a pivotal role in intervertebral disc degeneration, particularly under conditions of mechanical stress. Xu et al. demonstrated that intermittent cyclic mechanical tension (ICMT) promotes CEP degeneration through activation of the Wnt/β-catenin and disruption of *E-cadherin/β-catenin* complexes (Xu et al., 2016; Xiao et al., 2022). Activation of this pathway has been shown to induce nucleus pulposus (NP) cell apoptosis and accelerate extracellular matrix (ECM) degradation by upregulating catabolic enzymes, including matrix metalloproteinases (MMPs), thereby exacerbating disc degeneration (Che et al., 2021; Xiang et al., 2024). Additionally, Wu et al. and Zhu et al. reported that Wnt/β-catenin activation contributes to NPs apoptosis and ECM degradation, exacerbating disc degeneration (Wu et al., 2022; Zhu et al., 2022). In the clinical context, pharmacological activation of Wnt signaling has been widely explored for the prevention and treatment of osteoporosis (Oh et al., 2023), primarily aiming to enhance bone mineral density. However, emerging evidence suggested that sustained activation of this pathway may promote calcification of the cartilaginous endplate (Sun and Luo, 2019). Such calcific changes might impede the transport of nutrients and oxygen to the central part of the intervertebral disc, thereby accelerating degenerative processes and contributing to IVDD progression (Sun and Luo, 2019; Zehra et al., 2022).

Sclerostin (SOST), a glycoprotein predominantly secreted by osteocytes, is a well-characterized endogenous antagonist of the Wnt/β-catenin signaling pathway. Its expression is highly sensitive to mechanical stimuli and is downregulated under mechanical loading conditions (Robling et al., 2008). In addition, pro-inflammatory cytokines can also modulate its expression (Heiland et al., 2010), implying that *SOST* can regulate bone remodeling induced by both mechanical loading and inflammation (Tu et al., 2012). Given its inhibitory effect on Wnt signaling and its mechanosensitive nature, SOST may serve as a crucial mediator linking mechanical microenvironmental changes to molecular pathways involved in cartilaginous endplate degeneration.

Given that activation of the Wnt/β-catenin signaling pathway promotes degeneration and that SOST acts as a mechanosensitive inhibitor of this pathway, we postulated that excessive or sustained mechanical loading may downregulate SOST expression in CEP cells. This would lead to aberrant activation of Wnt/β-catenin signaling, thereby triggering matrix degradation and pathological calcification. Based on this, we turned our attention to ESWT, a non-invasive mechanical stimulus with proven regenerative effects in bone and cartilage tissues, as a potential intervention. We hypothesized that ESWT could modulate the mechanical microenvironment of the CEP by upregulating SOST expression, thereby suppressing the Wnt/β-catenin pathway activation and attenuating CEP degeneration.

Building upon our previous findings, this study aims to investigate whether aberrant mechanical stress induces CEP degeneration through dysregulation of the SOST/Wnt/β-catenin signaling axis. Using a rat tail vertebral mechanical degeneration model and isolated CEP chondrocytes, we systematically examined the effects of mechanical pressure on CEP cell calcification and matrix degeneration at both cellular and molecular levels. Moreover, we evaluated whether ESWT could reverse or attenuate these degenerative alterations by modulating SOST expression and downstream Wnt/β-catenin signaling, both *in vitro* and *in vivo*. Collectively, our work seeks to provide new mechanistic insights into CEP degeneration and to establish a theoretical foundation for ESWT as a non-invasive therapeutic strategy for intervertebral disc degeneration.

## Materials and methods

### Experimental animals

12-week-old male Sprague-Dawley (SD) rats were obtained from the Animal Center of Soochow university, Suzhou, China (License No. SCXK [Su] 2019-0008). All rats were housed under standard laboratory conditions with *ad libitum* access to standard chow and water. Environmental parameters were maintained at a temperature of 25 °C ± 2 °C and a humidity range of 45%–70%, under a 12:12-h light-dark cycle (lights on from 9:00 a.m. to 9:00 p.m.). All experiments procedures were approved by the Animal Experimental Management Committee of our university (Approval No. SUDA20230911A01) and conducted in accordance with the ARRIVE criteria (Kilkenny et al., 2010).

### Animal modeling and grouping

A total of 40 male SD rats (average weight 250 ± 15 g) were included in this study. Upon arrival, all animals underwent a 7-day acclimatization period under standard housing conditions to minimize stress from transportation and environmental changes. According to our previous research experience, the rats were randomly allocated into four groups based on factors such as age and body weight (n = 10 per group): Control group, Model group, Self-repair after degeneration group (nESWT), and ESWT intervention group (ESWT).

Following our previously established and extensively validated research protocols regarding the rat tail mechanical compression model (Che et al., 2021; Che et al., 2022; Che et al., 2019a; Guo et al., 2020), a mechanical compression-induced intervertebral disc degeneration model was established (Supplementary Figure S1). Briefly, general anesthesia was induced by intraperitoneal injection of pentobarbital sodium (50 mg/kg). Under X-ray guidance, sterilized Kirschner wires were inserted percutaneously into the rat tail vertebrae at levels Co7-Co10 using custom-designed molds to ensure accurate positioning. After radiographic confirmation of proper placement, a custom external fixation device was mounted on both sides of the tail. For the target segment (Co8-Co9), a continuous compression force of 1 mm was applied for exactly 4 weeks. Based on prior biomechanical validations, this 1 mm displacement generates approximately 1.0–1.5 MPa of compressive stress, which adequately simulates the chronic mechanical overloading relevant to human IVDD pathology (Che et al., 2021; Guo et al., 2020; Zhuo et al., 2021). To stabilize the construct, adjacent segments (Co7-Co8 and Co9-Co10) were fixed under 1 mm distraction. In the Control group, only Kirschner wires were inserted into the target vertebral segment without any fixation device. In the Model group (c-4w), rats were sacrificed immediately after the 4-week continuous compression to assess the degree of degeneration. In the nESWT group, following the 4-week mechanical loading, the external fixation device was removed, and no further intervention was applied for an additional 4 weeks, allowing for natural recovery. In the ESWT group, rats received Extracorporeal Shockwave Therapy (BTL-5000 SWT) at parameters of 1.5 Bar, 5 Hz, and 500 pulses intervention once a week for four consecutive weeks after the initial 4-week compression. These specific ESWT parameters were selected based on our previous findings demonstrating that low-energy shockwaves effectively promote cartilage matrix synthesis and tissue regeneration without inducing mechanical micro-damage to the structural integrity (Che et al., 2021; Guo et al., 2020).

### Measurement of intervertebral disc height and magnetic resonance imaging (MRI) analysis

At each designated time point, intervertebral discs morphology was evaluated using X-ray and MRI. X-rays were analyzed by means of a DR-X-ray machine (SHIMADZU, RAD SPEED M) with an exposure time of 10 s and 26 kV to obtain X-rays. The measurement of the intervertebral disc gap followed the method described by Masuda et al. (Masuda et al., 2005). Briefly, the disc height index (DHI) was calculated from radiographs using the formula: DHI = 2 × (anterior, middle, and posterior disc heights)/(anterior, middle, and posterior heights of the adjacent cranial and caudal vertebral bodies). The percentage DHI (%DHI) was normalized and expressed as (post-intervention DHI/pre-intervention baseline DHI) × 100.

MRI scans (GE HDE) were conducted using the FRFSE - XL scan sequence in the sagittal plane. Non-fat-saturated scan parameters were as follows: repetition time: 3,000 ms; echo time: 80 ms; field of view: 200 mm × 200 mm; slice thickness: 1.4 mm. The acquired images were assessed using the Pfirrmann grading system by three MRI physicians and three spinal surgeons.

### Histological analysis

At the end of the experimental period, rats were deeply anesthetized via intraperitoneal injection of sodium pentobarbital (50 mg/kg body weight) and subsequently euthanized by cervical dislocation. The Co8-9 intervertebral discs segments were carefully harvested and immediately fixed in 4% paraformaldehyde at room temperature for 24 h. Specimens were subsequently decalcified in 15% ethylenediaminetetraacetic acid (EDTA) solution for 4 weeks, with the decalcification solution replaced regularly to ensure complete mineral removal.

Following confirmation of complete decalcification, surrounding soft tissues were trimmed, and the samples were subjected to routine histological processing, including washing, graded ethanol dehydration, transparency enhancement, paraffin embedding, and sectioning. Afterward, Paraffin sections were stained with Hematoxylin and Eosin (H&E, Cat: C0105S, Beyotime, China) and Fast Green/Safranin O staining (Cat: C0621S, Beyotime, China) according to the manufacturer’s instructions. Stained sections were observed under a light microscope (XSP-2CA, Shanghai, China). To comprehensively and accurately assess the structural and compositional degenerative changes, histological morphology was evaluated using a standardized grading system established by Han et al. (Han et al., 2008). The detailed evaluation criteria of this scoring system are explicitly provided in Table 1. All analyses were performed independently by three experienced researchers in a blinded manner to minimize observational bias.

**TABLE 1:** Comprehensive histological grading system for evaluating intervertebral disc degeneration.

Category	Description	Score
Cellularity of the annulus fibrosus (AF)	Fibroblasts constitute >75% of the total cells	1
	Neither fibroblasts nor chondrocytes exceed 75% of the total cells	2
	Chondrocytes constitute >75% of the total cells	3
Morphology of the annulus fibrosus (AF)	Collagen lamellae are well-organized with no ruptures	1
	Presence of inward bulging, ruptured, or serpentine fibers	2
	Distinct and severe inward bulging, ruptured, or serpentine fibers	3
Boundary between AF and NP	Normal, intact interface without any interruptions	1
	Mild interruptions at the interface	2
	Moderate or severe interruptions at the interface	3
Cellularity of the nucleus pulposus (NP)	Normal cellularity with typical stellate-shaped cells	1
	Mild reduction in cell number; presence of chondrocyte-like cells	2
	Significant reduction in cell number (>50% decrease)	3
Morphology of the nucleus pulposus (NP)	Round shape, occupying at least half (>50%) of the disc area	1
	Round or irregular shape, occupying a small portion of the disc area	2
	Irregular shape, occupying less than one-fourth (<25%) of the disc area	3

The total histological degeneration score for each intervertebral disc is calculated as the sum of the scores from all five distinct categories. The total score ranges from 5 to 15, where 5 indicates a normal, healthy disc, and 15 indicates severe, end-stage intervertebral disc degeneration. Abbreviations: AF, annulus fibrosus; NP, nucleus pulposus. Grading criteria are adapted from the standardized system established by Han et al. (2008).

### Immunohistochemistry (IHC) staining

For IHC analysis, the decalcified paraffin-embedded sections were deparaffinized, rehydrated, and subjected to antigen retrieval. Endogenous peroxidase activity was blocked, followed by overnight incubation at 4 °C with primary antibodies against SOST and Collagen II. Subsequently, sections were incubated with an HRP-conjugated secondary antibody and visualized using DAB. Hematoxylin was used as a counterstain. The percentage of positive cells was quantified using ImageJ software.

## Isolation, culturing, mechanical loading, and shockwave intervention of CEP cells

Primary CEP cells were isolated from the coccygeal vertebrae (Co7-Co10) of male SD rats. Animals were anesthetized with sodium pentobarbital and euthanized prior to tissue harvesting. Tails were aseptically removed, skinned, and briefly immersed in 75% ethanol for surface sterilization. Subsequently, the vertebral segments were transferred to a biosafety cabinet and rinsed twice with phosphate-buffered saline (PBS) containing 1% penicillin-streptomycin (P/S). Under a stereomicroscope, the nucleus pulposus and annulus fibrosus were carefully removed. The thin, semi-transparent layer of cartilage immediately adjacent to the inner annulus fibrosus and nucleus pulposus—representing the true cartilaginous endplate (CEP)—was meticulously scraped off using a surgical scalpel. Extreme caution was exercised to strictly avoid the deeper epiphyseal growth plate cartilage and subchondral bone. CEP tissues were carefully dissected from the vertebral endplates. Specifically, based on the standardized micro-dissection protocol established in our previous studies focusing on the CEP mechanical microenvironment (Xiang et al., 2024), the thin, semi-transparent layer of cartilage immediately adjacent to the inner annulus fibrosus and nucleus pulposus was meticulously scraped off using a surgical scalpel under a stereomicroscope. Extreme caution was continuously exercised to strictly avoid the deeper epiphyseal growth plate cartilage and subchondral bone. The isolated tissues were then digested in 0.2% type II collagenase at 37 °C for 5–6 h with gentle agitation. After enzymatic digestion, the cell suspension was filtered through a 100 μm cell strainer and centrifuged at 1,200 rpm for 5 min. The supernatant was discarded, and the cell pellet was washed twice with PBS and centrifuged again. Cells were resuspended in DMEM/F12 complete medium containing 10% fetal bovine serum (FBS) and 1% P/S and cultured in standard conditions (37 °C, 5% CO_2_). The culture medium was renewed every other day. Upon reaching approximately 90% confluence, cells were passaged, and used for subsequent *in vitro* experiments.

To establish an *in vitro* mechanical compression model, flexible-bottom culture dishes were pre-stretched to 10% deformation using a mechanical loading device (Supplementary Figure S1). CEP cells were seeded onto the pre-stretched dishes at a density of 3,000 cells/cm^2^. When cells reached 80%–90% confluence, the stretching force applied to the dishes was released, allowing the culture surface to return to its original state and thereby generating compressive mechanical stress on the attached cells.

Following 5 days of cyclic compressive loading (10% deformation) to induce mechanical stress-associated degeneration, cells were subjected to ESWT under the parameters of 1.5 bar, 5 Hz, 500 pulses. After ESWT intervention, the cells were immediately reseeded into six-well plates and cultured for an additional 2 days before harvesting for subsequent Western blot and RT-PCR analyses.

### Cell viability assay

Cell viability was assessed using the Cell Counting Kit-8 (CCK-8, Cat: C0037, Beyotime, China) according to the manufacturer’s instructions. Briefly, on days 1, 3, 5, and 7 after seeding, 10% CCK-8 working solution was added directly to each stretching dish, and the cells were incubated at 37 °C for 2 h. Subsequently, the supernatant was transferred to a 96-well plate, and the absorbance at 450 nm was measured using a microplate reader (Molecular Devices, United States).

### RT-PCR analysis of gene expression

Total RNA was extracted from each sample using the TRIzol® reagent (Cat: 15596018, Thermo Fisher Scientific, United States), and 1 μg of total RNA was reverse transcribed into cDNA using the reverse transcription first-strand cDNA synthesis kit (Cat: K1612, Thermo Fisher Scientific, United States). RT-qPCR was performed using the iTaqTM universal SYBR® green supermix kit (Cat: 1725121, Bio-Rad, United States). For each reaction, cDNA corresponding to 50 ng of total RNA was used as the template. The mRNA expression levels of t*ype II collagen, aggrecan, MMP3, MMP13, Runx2, OCN, SOST,* and *β-catenin* were detected. GAPDH served as an internal standard, and the primer sequences enlisted in Supplementary Material (Table 2)**.**

**TABLE 2:** Primer sequences used for quantitative real-time PCR.

Gene	Primer sequence (5′–3′)	
RUNX2	Forward Reverse	AGCGGACGAGGCAAGAGTTT CTGTCTGTGCCTTCTTGGTTCC
SOST	Forward Reverse	TGCCCTTGCCAGTGCTTCC CGCTGTGACCACGGTGATTTT
Aggrecan	Forward Reverse	CAAGTCCCTGACAGACACCC GTCCACCCCTCCTCACATTG
Collagen II	Forward Reverse	ATCGCCACGGTCCTACAATG GGCCCTAATTTTCGGGCATC
β-catenin	Forward Reverse	ACTCCAGGAATGAAGGCGTG GAACTGGTCAGCTCAACCGA
OCN	Forward Reverse	GCAGACCTAGCAGACACCAT TTGGACATGAAGGCTTTGTCA
MMP3	Forward Reverse	TTTGGCCGTCTCTTCCATCC GCATCGATCTTCTGGACGGT
MMP13	Forward Reverse	ACCATCCTGTGACTCTTGCG TTCACCCACATCAGGCACTC
GAPDH	Forward Reverse	AGTGCCAGCCTCGTCTCATA GATGGTGATGGGTTTCCCGT

### Western blot analysis

Total protein was extracted from CEP cells using a lysis buffer containing 1% protease inhibitor (Cat: P6730, Solarbio, China). Cells were lysed on ice for 30 min and subsequently centrifuged to obtain the clarified supernatant. Protein concentration was measured using a BCA protein assay kit (Cat: P0012, Biyotime, China). Equal amounts of protein samples (30 μg) were separated by 10% sodium dodecyl sulfate-polyacrylamide gel electrophoresis (SDS-PAGE) and transferred onto nitrocellulose membranes (Cat: FFN53, Beyotime, China).

The membranes were blocked with a 5% non-fat milk (Cat: P0216, Biyotime, China) in TBST at room temperature for 2 h. The membranes were incubated overnight at 4 °C with primary antibodies against *Runx2* (Cat: 20700-1-AP, Proteintech, China), *OCN* (Cat: DF12303, Affinity, China), *β-catenin* (Cat: 51067-2-AP, Proteintech, China), *Collagen II* (1:1000, Proteintech, China), *Aggrecan* (Cat: 13880-1-AP, Proteintech, China), *MMP3* (Cat: 17873-1-AP, Proteintech, China), *MMP13* (Cat: 18165-1-AP, Proteintech, China), *SOST* (1:1000, Proteintech, China), and *Tubulin* (Cat: AF2827, Beyotime, China), which served as the loading control.

After washing with TBST, membranes were incubated with horseradish peroxidase (HRP)-conjugated secondary antibodies at room temperature for 1 h, including HRP-conjugated goat anti-rabbit IgG (Cat: A0208, Beyotime, China) and HRP-conjugated goat anti-mouse IgG (Cat: A0216, Beyotime, China), according to the host species of the primary antibodies. After that, protein bands were visualized using enhanced chemiluminescence substrate (SuperSignal West Pico Substrate, Cat: 34579, Thermo Fisher Scientific, United States) and detected with a Bio-Rad ChemiDoc Imaging System. Band intensities were quantified using ImageJ software (National Institutes of Health, Bethesda, MD, United States).

The original, uncropped images of all Western blot membranes, including the locations of molecular weight markers, are provided in Supplementary Figure S2.

#### Cell transfection

A *SOST* overexpression plasmid was designed and constructed by GenePharma Co., Ltd (Suzhou, China). Second-passage CEP cells were seeded onto pre-stretched dishes at an appropriate density. Transient transfection was performed using Lipofectamine 3000. Briefly, the SOST overexpression plasmid and Lipofectamine 3000 reagent were diluted separately in serum-free medium and then combined at the recommended ratio to form DNA-lipid complexes. The mixture was gently mixed and incubated at room temperature for 20 min to allow complex formation.

The transfection complexes were added to the cells and gently mixed, and incubated at 37 °C with 5% CO_2_ in a cell culture incubator for 6 h, after which the culture medium was replaced with fresh complete medium. Transfected cells were cultured for an additional 48 h before subsequent experiments were performed.

### Immunofluorescence staining

CEP cells cultured on stretching dishes were fixed with 4% paraformaldehyde for 15 min. Then, the elastic membranes were carefully cut to an appropriate size using corresponding molds and transferred into 24-well plates for subsequent staining procedures. Cells were treated with a permeabilization solution (Cat: P0096, Beyotime, China) for 10 min. After washing with PBS, cells were blocked with blocking buffer (Beyotime, China) for 30 min to reduce nonspecific binding. Cells were then incubated overnight with appropriately diluted primary antibodies against β-catenin and SOST. Following three washes with PBS, cells were incubated with Alexa Fluor® 647-conjugated secondary antibody (ab150075, Abcam, United States) for 1 h at room temperature in the dark. For cytoskeleton visualization, cells were stained with Actin-Tracker Green-488 (Cat: C2201S, Beyotime, China; 1:200 dilution). Cell nuclei were counterstained with 4′,6-diamidino-2-phenylindole (DAPI, Cat: C1002, Beyotime, China). Fluorescence images were captured using a Zeiss Axiovert 40CFL microscope (Zeiss, Oberkochen, Germany). The quantitative analysis of fluorescence intensity for β-catenin and SOST was performed using ImageJ software.

### Scanning electron microscopy (SEM) evaluation of the bony endplate

SEM was used to observe the micro- and nanoscale morphology of the bony endplate, following the procedure described in our prior study (Che et al., 2019b). After euthanasia, rat tail vertebrae were harvested and surrounding soft tissues were carefully removed. The specimens were subjected to enzymatic digestion to eliminate residual soft tissue, followed by graded ethanol dehydration and air drying. The samples were then placed in a cool, well-ventilated environment for 24 h. During this process, the bony endplate naturally separated from the vertebral body, consistent with our previously established method.

The naturally dried endplate samples were further dehydrated using a critical point dryer. Using conductive adhesive, the samples were fixed onto metal sample holders and then uniformly coated with a thin layer of gold using an ion sputter coater. The dried samples and the coated sample holders were subjected to SEM observation.

For quantitative analysis, a standardized region of interest (ROI, 1 × 1 mm^2^) was strictly defined in the central area of the bony endplate. Within this ROI, pore density was defined as the number of pores per square millimeter. Pore length (diameter) was determined by measuring the average maximum diameter of the identified pores using ImageJ software (version 1.54p, Java 1.8.0_452).

### Statistical analysis

Data analysis and graphical representations were performed using GraphPad Prism 6 (GraphPad Software, United States) and SPSS 24.0 (IBM SPSS Inc., Chicago, IL, United States) statistical software. Experimental data were presented as mean ± standard deviation. Comparisons between two groups were performed using Student’s t-test. Comparisons among multiple groups were performed using one-way ANOVA followed by Tukey’s *post hoc* test. Data were analyzed using two-way ANOVA to assess the main effects of treatment and time and their interaction, followed by Tukey’s multiple comparisons test. Homogeneity of variances was assessed for all data. A value of *P ≤ 0.05* was considered to be statistically significant.

## Results

### ESWT improves disc height and T2 signal intensity in degenerated intervertebral discs

**FIGURE 1:**
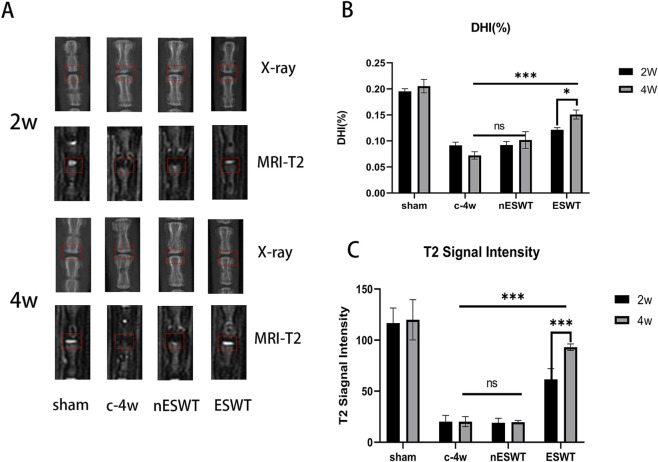
ESWT restores disc height and hydration *in vivo*. **(A)** Representative X-ray and MRI T2-weighted images showing intervertebral disc morphology and signal intensity in rat tail vertebrae following ESWT intervention (highlighted with a red wire frame). **(B,C)** Quantitative analysis of disc height index (DHI) and MRI-T2 signal intensity. Comparisons were performed using two-way ANOVA followed by Tukey’s *post hoc* test. *P ≤ 0.05, **P ≤ 0.01, ***P ≤ 0.001. The “sham” and “c-4w” groups represent the baseline control and the 4-week compression model without ESWT, respectively. The 2-week and 4-week time points represent selected stages post-intervention (i.e., 2 and 4 weeks after the removal of the external fixation device) to dynamically monitor disease progression and recovery status for both the natural recovery (nESWT) and ESWT-treated groups.

To evaluate the therapeutic effects of ESWT on degenerated intervertebral discs *in vivo*, radiographic and MRI analyses were performed to assess structural restoration. Radiographic analysis demonstrated that disc height was significantly increased in the ESWT group compared to the model group (c-4w) at both 2 and 4 weeks post-intervention (following the removal of the external fixation device), with the 4-week time point showing greater improvement than 2 weeks (Figure 1A). Quantitative analysis of the Disc Height Index (DHI) further confirmed these findings (Figure 1B). At 2 weeks, the ESWT group showed a significantly higher DHI compared with the nESWT group (*P* = 0.0422). By 4 weeks, this difference became more substantial (*P* = 0.0006). Furthermore, within the ESWT group, DHI values at 4 weeks were significantly higher than those at the post-injury baseline (c-4w) (*P* = 0.0003), demonstrating progressive structural recovery over time. Consistently, T2-weighted MRI images demonstrated enhanced signal intensity in the ESWT group, indicating improved hydration and disc integrity relative to both the model and nESWT groups (Figure 1C).

### ESWT promotes structural restoration of bone endplate and intervertebral disc histology

**FIGURE 2:**
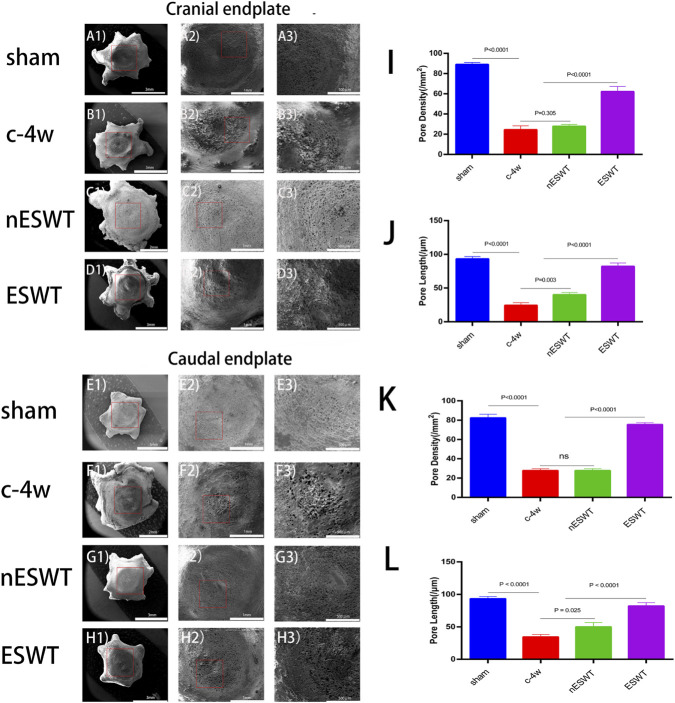
ESWT improves vertebral endplate microstructure. **(A–H)** Scanning electron microscopy (SEM) images of cranial **(A–D)** and caudal **(E–H)** bony endplates in four groups: Sham (A1–3, E1–3), c-4w (B1–3, F1–3), nESWT (C1–3, G1–3), and ESWT (D1–3, H1–3). Each set includes overall and locally magnified views. The red square regions in the overall views define the standardized region of interest (ROI, 1 × 1 mm^2^) utilized for the quantitative analysis. **(I,J)** Quantitative analysis of cranial endplate pore density **(I)** and pore diameter **(J)**. **(K,L)** Quantitative analysis of caudal endplate pore density **(K)** and pore diameter **(L)**. Statistical comparison shows significant improvements in the ESWT group compared to c-4w group (*P <* 0.0001). *n* = 7 each group. Comparisons among multiple groups were performed using one-way ANOVA followed by Tukey’s *post hoc* test.

Scanning electron microscopy (SEM) was performed to evaluate ultrastructural changes in the bony endplates following intervention (Figures 2A–H). In the sham group, both cranial and caudal endplates exhibited abundant irregular pores with a typical “concave” morphology, high pore density, and moderate pore diameter (Figures 2A,E). In contrast, after 4 weeks of axial compression (c-4w group), marked structural deterioration was observed in both cranial (Figure 2B) and caudal endplates (Figure 2F). Pore density and diameter were significantly reduced (Figures 2I–L), accompanied by pronounced calcification, sclerosis, and bone hypertrophy (defined herein as the abnormal thickening and increased density of the bony trabeculae in the subchondral bone, distinct from chondrocyte hypertrophy). Degenerative changes were more severe in the central region than in the periphery region, and the caudal endplate exhibited greater structural damage. In the nESWT group, limited spontaneous recovery of pore morphology was observed. Although bone hypertrophy persisted (Figures 2C,G), pore diameter was significantly increased compared with the c-4w group (Figures 2J,L). However, improvements in pore density and sclerosis were minimal (Figures 2I,K). In contrast, the ESWT group demonstrated substantial restoration of endplate architecture in both cranial (Figure 2D) and caudal (Figure 2H) endplates. ESWT significantly increased pore density and diameter (Figures 2I–L) and alleviated bone hypertrophy and sclerosis. Compared with the nESWT group, ESWT induced more pronounced structural recovery of the osseous endplates.

**FIGURE 3:**
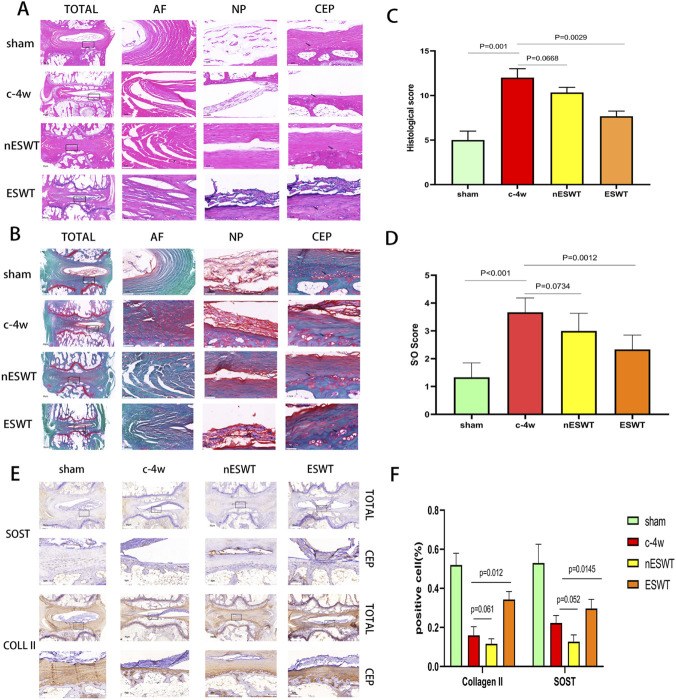
Histological evaluation of the intervertebral disc tissues following ESWT intervention. **(A)** Hematoxylin/eosin (H&E) staining of intervertebral discs in each group. **(B)** Safranin O-Fast Green staining of each group, highlighting proteoglycan content and cartilage structure. Black bounding boxes in the “TOTAL” column indicate the regions of interest magnified in the subsequent columns. Black arrows specifically indicate the thin layer of the true cartilaginous endplate (CEP). **(C)** Histological score indicating a significant statistical difference between the ESWT group and c-4W group (*P* = 0.0029). **(D)** Semi-quantitative analysis of Safranin O-Fast Green staining score showing a significant difference observed between the ESWT group and c-4w group (*P* = 0.0012). **(E)** Representative immunohistochemistry (IHC) images of SOST and Collagen II (COLL II) in the cartilaginous endplates. **(F)** Quantitative analysis of the percentage of positive cells for Collagen II and SOST. *n* = 7 each group. Comparisons among multiple groups were performed using one-way ANOVA followed by Tukey’s *post hoc* test.

H&E staining revealed distinct structural differences among groups (Figure 3A). In the sham group, the intervertebral disc (IVD) maintained normal morphology, characterized by abundant and orderly arranged CEP cells, well-preserved NPs, and intact annulus fibrosus architecture. In contrast, the c-4w group exhibited severe histological degeneration. The number of cartilaginous endplate cells was markedly reduced, and their arrangement shifted from an orderly pattern to clustered aggregation. Additionally, there was a noticeable reduction in the NPs, and the annulus fibrosus exhibited more prominent disruptions. Both nESWT and ESWT groups exhibited varying degrees of histological improvement compared with the c-4w group. In the nESWT group, there was a slight increase in cartilaginous endplate cell count, but the improvement in nucleus pulposus cells and annulus fibrosus was not significant, indicating that the self-healing ability of rats in the face of degeneration was limited. In the ESWT group, the cartilaginous endplate layer was notably thicker. Specifically, the cartilaginous endplate cells—identified by their typical rounded chondrocytic morphology residing within distinct lacunae—were significantly increased in count and exhibited a more orderly arrangement compared to the c-4w group. There were also varying degrees of improvement in the nucleus pulposus and annulus fibrosus.

Safranin O-Fast Green staining further confirmed these findings (Figure 3B). The sham group exhibited intense Safranin O staining, indicating abundant proteoglycan content within the CEP. The c-4w group showed markedly reduced staining intensity, reflecting severe matrix degradation. The nESWT group displayed partial restoration of proteoglycan content but retained noticeable defects. In contrast, the ESWT group demonstrated markedly enhanced staining intensity, indicating improved matrix synthesis and structural recovery of the CEP.

Quantitative histological scoring (Figures 3C,D) supported the above observations. Both H&E-based and Safranin O-based scores were significantly elevated in the c-4w group compared to the sham group (*P* < 0.001), reflecting severe degeneration. While the nESWT group showed a mild reduction in scores compared to the model group (*P* = 0.0668 and *P* = 0.0734, respectively), the ESWT group exhibited a significant improvement (*P* = 0.0029 and *P* = 0.0012), outperforming the nESWT group and indicating superior therapeutic efficacy of ESWT in restoring disc histology and matrix composition.

To further validate our mechanism *in vivo*, we performed IHC staining for SOST and Collagen II in the cartilaginous endplates (Figure 3E). Consistent with our histological findings, the percentage of SOST and Collagen II positive cells was significantly decreased in the c-4w group compared to the sham group. Importantly, ESWT intervention markedly restored the expression of both SOST and Collagen II in the degenerated endplate tissues (Figure 3F), corroborating our *in vitro* mechanistic data.

### Compressive stress induces degeneration of CEP cells *in vitro*

**FIGURE 4:**
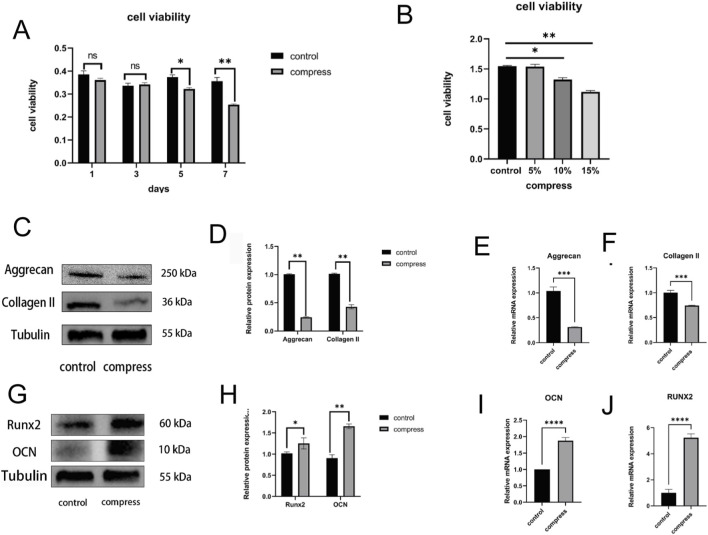
Compressive stress induces degeneration in CEP chondrocytes. **(A,B)** Cell viability assessed by CCK-8 assay **(A)** Time-dependent effects over 1, 3, 5, and 7 days under 10% compressive stress. **(B)** Magnitude-dependent effects at 5%, 10%, and 15% compressive stress for 5 days. **(C, G)** Representative Western blot images showing the protein expression of chondrogenic markers (Aggrecan, Collagen II) and calcification factors (Runx2, OCN). **(D, H)** Quantification of relative protein expression levels from Western blots, normalized to Tubulin. **(E,F,I, J)** Relative mRNA expression levels of Aggrecan, Collagen II, OCN, and RUNX2 assessed by RT-PCR, normalized to GAPDH. *n* = 3 each group. Comparisons between two groups were performed using Student’s t-test. ns: not significant. *P ≤ 0 0.05, **P ≤ 0 0.01, ***P ≤ 0 0.001, ****P ≤ 0 0.0001. Original uncropped Western blot images are available in Supplementary Figure S2.

To investigate the effects of mechanical compressive on CEP chondrocytes, cells were subjected to cyclic compressive loading at varying intensities (5%, 10%, and 15%) and durations *in vitro*. The CCK-8 assay revealed that cell viability progressively decreased with increasing stress intensity and prolonged stimulation time (Figures 4A,B), indicating a stress magnitude- and time-dependent inhibitory effect on CEP cell survival.

To further evaluate phenotypic alterations induced by compressive stress, the expression levels of chondrogenic markers (Aggrecan, Collagen II) and calcification-related factors (Runx2, OCN) were examined by Western blot and RT-PCR. Results showed that compressive stress significantly downregulated *Aggrecan* and *Collagen II* expression (Figures 4C–F), while upregulated *Runx2* and *OCN* expression (Figures 4G–J). These findings suggest that prolonged or high-intensity compressive stress induces calcification and degeneration in CEP cells.

### Compressive stress downregulates SOST and activates the Wnt/β-catenin pathway in CEP cells

**FIGURE 5:**
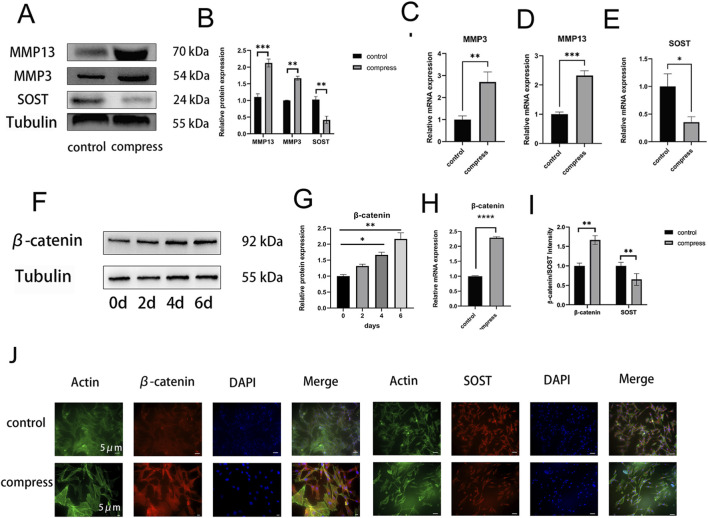
Compressive stress activates Wnt/β-catenin signaling and suppresses SOST expression in endplate chondrocytes. **(A)** Representative Western blot images of MMP13, MMP3, and SOST. **(B)** Quantification of protein levels from **(A)**, normalized to Tubulin. **(C–E)** Relative mRNA expression levels of MMP3, MMP13, and SOST assessed by RT-PCR, normalized to GAPDH. **(F)** Representative Western blot images showing the time-dependent accumulation of β-catenin (0, 2, 4, 6 days). **(G)** Quantification of β-catenin protein levels from **(F)**, normalized to Tubulin. **(H)** Relative mRNA expression of β-catenin assessed by RT-PCR. **(I)** Quantification of fluorescence intensity for β-catenin and SOST from the immunofluorescence images. **(J)** Representative immunofluorescence staining images of β-catenin (red/green) and SOST (red) in CEP cells. Scale bar = 5 μm. Note: Images in the compress group were deliberately captured with a larger field of view to better illustrate the widespread stress-induced pathological alterations in cell morphology and cytoskeletal arrangement. *n* = 3 each group. Statistical analysis for panel **(G)** was performed using one-way ANOVA followed by Tukey’s *post hoc* test. All other comparisons were analyzed using Student’s t-test. *P ≤ 0.05, **P ≤ 0.01, ***P ≤ 0.001, and ****P ≤ 0 0.0001. Original uncropped Western blot images are available in Supplementary Figure S2.

To explore the molecular mechanism driving CEP degeneration under mechanical stress, we examined the Wnt/β-catenin signaling pathway and its upstream inhibitor SOST. SOST, a secreted glycoprotein primarily expressed by osteocytes and CEP cells, acts as a potent negative regulator of Wnt signaling by binding to LRP5/6 receptors (Chang et al., 2014). Exposure of CEP cells to compressive stress resulted in a significant downregulation of SOST at both mRNA and protein levels (Figures 5A,B,E,I). This suppression of SOST may serve as a key initiating event in the activation of the Wnt/β-catenin pathway. Consistently, Western blot analysis showed a time-dependent accumulation of *β-catenin* protein in CEP chondrocytes following mechanical loading (Figures 5F,G), which was paralleled by an increase in β-catenin mRNA expression (Figures 5H,I). Immunofluorescence analysis further confirmed these findings, showing decreased SOST and elevated β-catenin protein accumulation in pressure-stressed CEP cells (Figure 5J). Together, these results suggest that stress-induced Wnt/β-catenin activation may be associated with suppression of SOST expression. Furthermore, compressive stress upregulated matrix metalloproteinases (*MMPs*) *MMP-3* and *MMP-13* at both the mRNA and protein levels (Figures 5A–D), indicating enhanced extracellular matrix breakdown as part of the degenerative response.

### Upregulation of SOST alleviates pressure-induced degeneration of CEP cells

To determine whether upregulation of SOST could attenuate CEP degeneration under mechanical stress, two strategies were employed: SOST gene overexpression and ESWT intervention.

**FIGURE 6:**
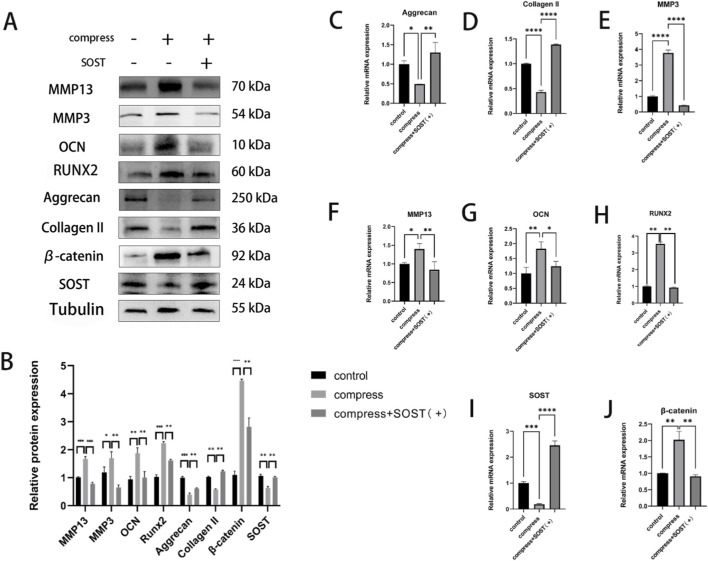
SOST overexpression alleviates degeneration in endplate chondrocytes under compressive stress. **(A,B)** Western blot analysis and quantification of Anabolic genes (Aggrecan, Collagen II), calcification factors (Runx2, OCN), catabolic genes (MMP3, MMP13), SOST, and β-catenin. **(C–J)** RT-PCR and quantification of anabolic genes (Aggrecan, Collagen II), calcification factors (Runx2, OCN), catabolic genes (MMP3, MMP13), SOST, and β-catenin. *n* = 3 each group. Comparisons among multiple groups were performed using one-way ANOVA followed by Tukey’s *post hoc* test. *P ≤ 0 0.05, **P ≤ 0.01, ***P ≤ 0.001, ****P ≤ 0 0.0001. Original uncropped Western blot images are available in Supplementary Figure S2.

*SOST* overexpression was achieved via Lipofectamine 3000 -mediated transfection in endplate chondrocytes. Western blot and RT-PCR analyses demonstrated that SOST-overexpressing chondrocytes exhibited significantly higher levels of chondrogenic markers (Aggrecan, Collagen II) and reduced expression of catabolic and osteogenic markers (Runx2, OCN, MMP-3, MMP-13) compared with the pressure-only group (Figures 6A–J), suggesting that SOST overexpression effectively mitigates pressure-induced degeneration.

**FIGURE 7:**
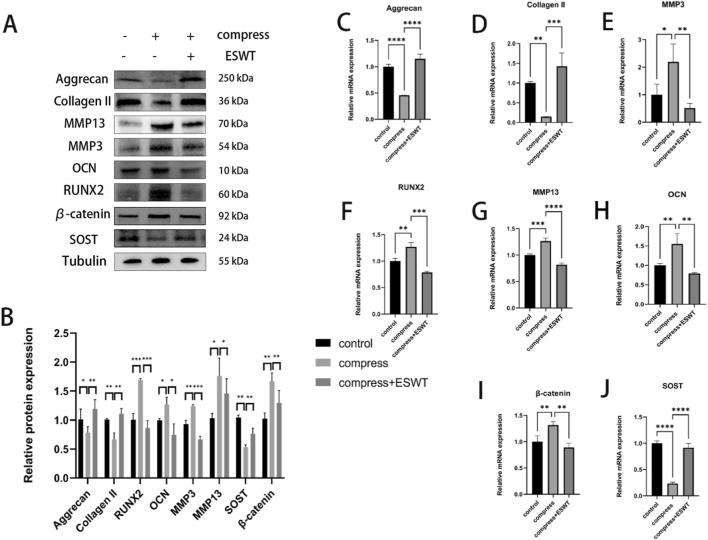
ESWT attenuates degeneration in endplate chondrocytes via modulation of SOST/Wnt/β-catenin signaling. **(A,B)** Western blot analysis and quantification of Aggrecan, Collagen II, calcification factors (Runx2, OCN), MMP3, MMP13, SOST, and β-catenin in degraded endplate chondrocytes with or without ESWT intervention. **(C–J)** RT-PCR analysis and quantitation of cartilage markers (Aggrecan, Collagen II), calcification factors (Runx2, OCN), MMP3, MMP13, SOST and β-catenin in degraded endplate chondrocytes with or without ESWT intervention. *n* = 3 each group. Comparisons among multiple groups were performed using one-way ANOVA followed by Tukey’s *post hoc* test. *P ≤ 0.05, **P ≤ 0.01, ***P ≤ 0.001, and ****P ≤ 0.0001. Original uncropped Western blot images are available in Supplementary Figure S2.

Similarly, application of ESWT (1.5 bar, 5 Hz, 500 pulses) to pressure-stressed CEP cells resulted in partial restoration of chondrogenic marker expression (*Aggrecan* and *Collagen II*), and significant downregulation of *Runx2*, *OCN*, *MMP-3* and *MMP-13* (Figures 7A,B). RT-PCR results further confirmed that ESWT can delay the degeneration of endplate chondrocytes induced by pressure (Figures 7C–J).

Collectively, these findings suggest that both SOST overexpression and ESWT-mediated SOST upregulation can effectively inhibit Wnt/β-catenin pathway activation and attenuate CEP degeneration induced by mechanical stress.

## Discussion

In this study, we demonstrated that compressive stress induces degeneration of CEP cells by downregulating SOST and activating the Wnt/β-catenin signaling pathway. Mechanistically, suppression of SOST under mechanical loading appears to act as an early trigger, leading to β-catenin accumulation, upregulation of catabolic enzymes, and promotion of extracellular matrix degradation and calcification in CEP cells. Both SOST overexpression and ESWT effectively mitigated CEP degeneration by restoring SOST levels and inhibiting downstream Wnt/β-catenin activity. *In vivo*, ESWT effectively improved disc height and T2-weighted MRI signal intensity, and promoted histological recovery of both bony and cartilaginous endplates. These findings identify the SOST/Wnt/β-catenin axis as a critical mechanotransduction pathway in stress-induced CEP degeneration and highlight ESWT as a promising therapeutic strategy.

Biomechanical loading, transmitted through intracellular signaling, represents a key determinant of cartilage development and function (O'Conor et al., 2013). Our results indicate that CEP cells possess a limited tolerance to compressive loading. When mechanical deformation remained below approximately 10%, chondrocyte viability was relatively preserved. However, sustained or excessive compression led to marked reductions in the expression of chondrogenic markers (Aggrecan and Collagen II), accompanied by upregulation of calcification-associated genes (Runx2 and OCN). This highlights a critical stress threshold, beyond which CEP degeneration becomes irreversible. Collectively, these findings underscore the importance of mechanical homeostasis in preserving CEP function and delaying IVDD progression.

The Wnt/β-catenin signaling pathway serves as a critical regulator of cartilage development and is essential for maintaining the morphology and function of the IVD (Kondo et al., 2011). Mechanical stress has been shown to activate the Wnt/β-catenin signaling pathway through various mechanisms, including altering the stiffness and structure of the extracellular matrix, which affects receptor molecules on the cell membrane and influences the organization and dynamics of the cell cytoskeleton (Zhu et al., 2023; Shi et al., 2023). Notably, the activation of the Wnt/β-catenin signaling pathway is time-dependent, where moderate activation can inhibit apoptosis and senescence of NP cells and promote cellular autophagy. In contrast, excessive activation exerts detrimental effects (Li et al., 2019). Xu et al.'s study revealed that intermittent cyclic tensile stress results in the overactivation of the Wnt/β-catenin signaling pathway, contributing to CEP degeneration (Xu et al., 2016). Conversely, inhibiting the activation level of the Wnt/β-catenin signaling pathway can alleviate the tensile stress-induced differentiation of CEP cells (Xu et al., 2016).

Mechanistically, mechanical stress-induced CEP degeneration is closely associated with downregulation of SOST, a key inhibitor of the Wnt/β-catenin pathway. Our *in vitro* experiments demonstrated that suppression of SOST under stress conditions led to excessive accumulation of β-catenin and upregulation of downstream catabolic genes, which collectively disrupted ECM integrity. Conversely, SOST overexpression effectively dampened Wnt/β-catenin signaling, reduced matrix degradation, and preserved chondrogenic marker expression. Consistent with these findings, Tori Kroon et al. reported that reducing SOST/sclerostin enhances disc structure *in vivo* through activation of canonical Wnt signaling. Collectively, these results highlight SOST as a mechanosensitive regulator and a critical link between mechanical load and Wnt pathway-mediated CEP degeneration.

The ECM of the endplate cartilage provides a protective barrier for chondrocytes, shielding them from external mechanical pressures and mitigating the detrimental effects of various external stimuli (Roughley, 2004). Prior research indicates that the Wnt/β-catenin signaling pathway induces the expression of *MMPs* and *TGFβ*, promoting IVD cell senescence and ECM remodeling. Specifically, Wnt/β-catenin signaling induces *MMPs* expression in NP cells, enhancing the expression of *MMPs* mRNA and protein and consequent ECM degradation (Xu et al., 2016). In our previous studies (Che et al., 2021; Guo et al., 2020), ESWT has been identified as an exogenous physical-mechanical stimulus that governs gene expression and elicits tissue repair and regeneration (Walewicz et al., 2020). In earlier research (Che et al., 2021), it was found that LTT enhances IVD rehydration and actively reconstructs the BEP in synergy with low-energy ESWT. This is achieved by suppressing collagen degradation through downregulation of *MMP-3*, *MMP-13*, and *ADAMTS-4* expression, thereby promoting ECM regeneration. This approach reduces AF circumferential tension and NP stress, reshaping the biomechanical microenvironment necessary for IVD regeneration and repair. In the present study, we demonstrate that mechanical stress activates the Wnt/β-catenin pathway by downregulating SOST, promoting CEP degeneration. ESWT reverses this process by upregulating SOST, thereby enhancing Col II and Aggrecan expression while suppressing MMP-3 and MMP-13. Similarly, SOST overexpression exerts protective effects through the same pathway. These findings are consistent with previous research (Chang et al., 2018; Kroon et al., 2022). These findings suggest that ESWT reshapes the CEP microenvironment by modulating Wnt/β-catenin–mediated ECM metabolism, with MMP-3 and MMP-13 as key downstream effectors.

Calcification of the CEP and alterations in the subchondral bone (BEP) are hallmarks of IVDD, often manifesting as early as Pfirrmann grade III, suggesting their association with the early development of disc degeneration (Che et al., 2019b). Morphological changes in the vertebral endplate can impair nutrient diffusion and alter load distribution, accelerating disc degeneration. Our previous study confirmed that along with disc degeneration, particularly in Pfirrmann grades IV-V, vertebral endplates exhibit sclerosis, ossification, osteophyte formation, and pore occlusion (Che et al., 2019b). In this study, ESWT significantly restored disc height and hydration, improved endplate pore morphology and density, and was accompanied by decreased *Runx2* and *OCN* expression. Meanwhile, histological analysis further showed thickening of the cartilaginous endplate layer, increased cell counts, and structural improvement in the nucleus pulposus and annulus fibrosus. This suggests that ESWT intervention preserves or restores the endplate nutrient pathways and reshapes the degenerative microenvironment, in part by promoting chondrocyte proliferation under optimized mechanical stimulation (Ioppolo et al., 2014; Zhao et al., 2021).

IVDD and Osteoarthritis (OA) are common degenerative musculoskeletal disorders that share similar pathological features, including ECM degradation, subchondral bone sclerosis, and increased expression of MMPs and ADAMTSs (Walewicz et al., 2020). In both diseases, subchondral bone thickening impairs nutrient diffusion and elevates mechanical stress on adjacent cartilage, contributing to degeneration (Malinin and Ouellette, 2000; Radin et al., 1972). Recent studies have shown that ESWT can reverse subchondral osteosclerosis and promote cartilage regeneration by stimulating chondrocyte activity (Wa et al., 2017; Chou et al., 2019). Consequently, reversing subchondral osteosclerosis to delay or repair cartilage degeneration has emerged as a recognized target for ESWT-based OA treatment (Wa et al., 2017; Chou et al., 2019). Building on these parallels, our study applied ESWT *in vitro* and *in vivo* to degenerated endplates and demonstrated that it upregulates SOST expression, inhibits Wnt/β-catenin signaling, reduces MMP expression, and improves ECM metabolism. These findings suggest a shared therapeutic mechanism of ESWT in IVDD and OA and support its potential as a non-invasive strategy for disc regeneration.

There are several limitations to this study. It remains unclear whether the observed restoration of the nucleus pulposus and annulus fibrosus following ESWT is a direct result of the intervention or a secondary outcome resulting from the restoration of the degenerated cartilaginous (vertebral) endplate layers, which might lead to the re-establishment of nutrient pathways. Clarifying this causal relationship requires further long-term research observations with larger sample sizes and detailed mechanistic investigations. In addition, although our *in vitro* experiments demonstrated that compressive stress activates the Wnt/β-catenin pathway and induces extracellular matrix degradation in CEP cells, we did not perform extended time-course analyses to systematically evaluate temporal dynamics of pathway activation. Furthermore, while our *in vitro* results clearly demonstrated that mechanical stress activates the Wnt/β-catenin pathway and downregulates SOST, our *in vivo* validation using immunohistochemistry was limited to SOST and Collagen II. The lack of direct *in vivo* verification for downstream β-catenin protein expression within the rat tail model represents a limitation of the current study. Future *in vivo* investigations employing spatial transcriptomics or targeted protein assays are required to definitively map the complete SOST/Wnt/β-catenin signaling cascade within the mechanically loaded intervertebral disc. Previous studies have shown that mechanical loading induces time-dependent modulation of β-catenin signaling, with early activation followed by dynamic regulatory adjustments in mechanosensitive cells (Lara-Castillo et al., 2015; Case et al., 2008). Therefore, while our selected time points were based on established mechanical loading models, a more comprehensive temporal profiling of stress duration would provide deeper insight into the kinetics of Wnt/β-catenin activation and its downstream catabolic responses. This represents an important direction for future investigation.

## Conclusion

Compressive stress induces vertebral endplate degeneration, which is closely associated with the downregulation of SOST expression *in vivo*. Supported by our *in vitro* evidence, this downregulation likely leads to the overactivation of the Wnt/β-catenin signaling pathway. Modulating this axis, either by restoring SOST expression or by inhibiting downstream catabolic signaling, effectively attenuates CEP degeneration, reduces ECM degradation, and promotes structural repair. ESWT exerts these protective effects in both *in vitro* and *in vivo* models, highlighting its potential as a non-invasive therapeutic strategy for intervertebral disc degeneration. These findings provide a theoretical and experimental basis for developing SOST-targeted or ESWT-based interventions in the clinical management of early disc degeneration.

## Data Availability

The original contributions presented in the study are included in the article/Supplementary Material, further inquiries can be directed to the corresponding authors.

## Supplementary material

SUPPLEMENTARY FIGURE S1 Schematic diagram of the mechanical loading system. **(A)** Fixation of the rat tail vertebral segments using K-wires and aluminum alloy blocks to establish the mechanical compression model. The Co8-Co9 intervertebral disc was selected as the target experimental segment. **(B)** Compression loading auxiliary device used for pre-stretching preparation. **(C)** Mechanical loading unit used to apply controlled compressive force to disc segments. **(D)** Control unit for regulating amplitude, frequency and duration of mechanical loading.

SUPPLEMENTARY FIGURE S2 Original uncropped Western blot images. Original uncropped Western blot images for Figures 4, 5. In accordance with our laboratory’s standard protocol to evaluate multiple target proteins simultaneously and conserve reagents, the PVDF membranes were horizontally cut into specific molecular weight strips prior to blocking and primary antibody incubation. Therefore, the original scans represent these individual strips rather than full-length gels. Note: Molecular weight markers have been incorporated into the main figures to confirm protein specificity.
