# Supplementary figures and images for: ESWT attenuates compressive stress-induced cartilaginous endplate degeneration via modulation of the SOST/Wnt/β-catenin signaling pathway
Source: Front Bioeng Biotechnol. 2026 Jun 24;14:1769920. doi: 10.3389/fbioe.2026.1769920 (PMC13341607; doi:10.3389/fbioe.2026.1769920)

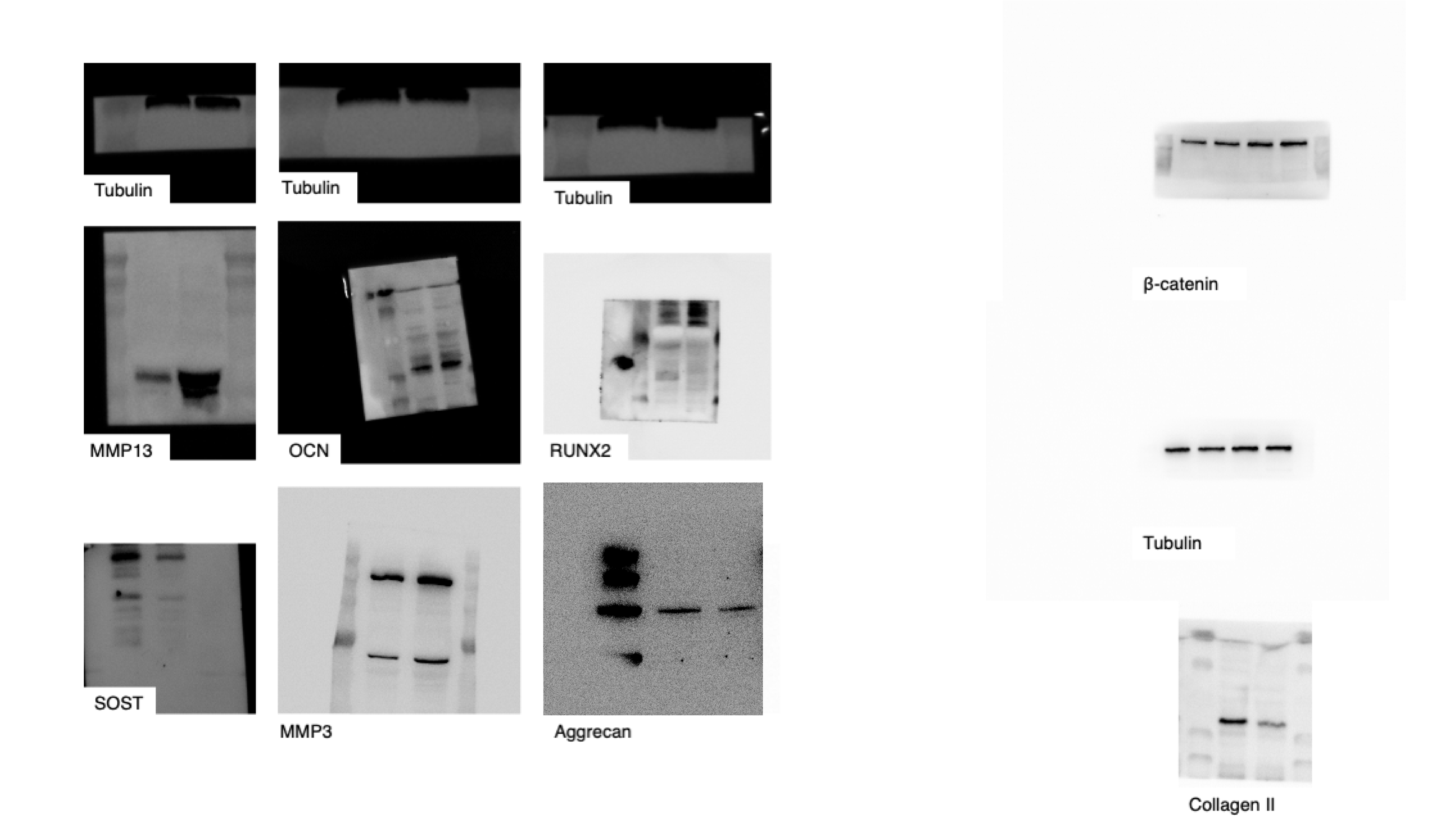

Supplement: Supplementary file 1 [file Image2.tif]

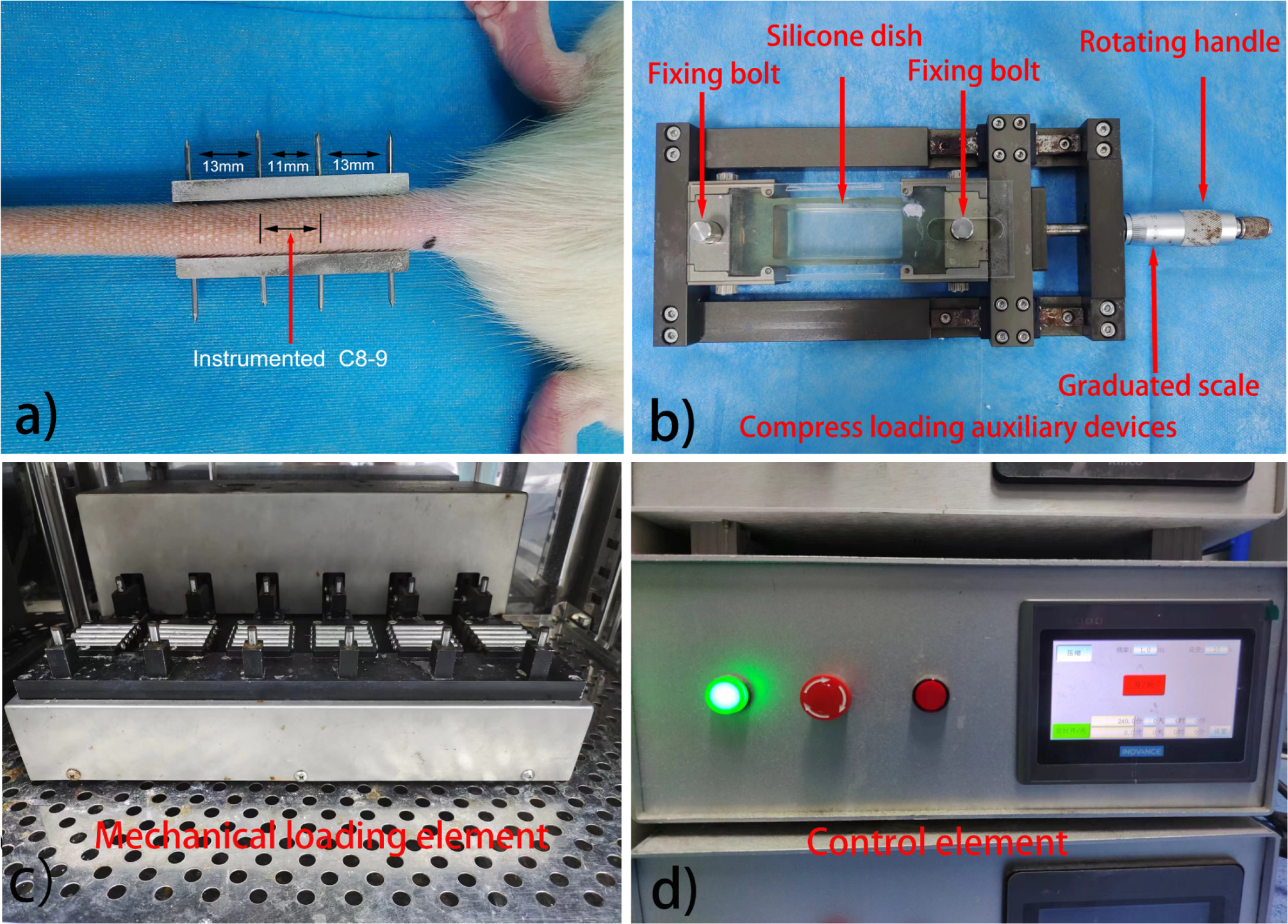

Supplement: Supplementary file 2 [file Image1.tif]
